# Effect of Resistance Exercise on Body Composition and Functional Capacity in Older Women with Sarcopenic Obesity—A Systematic Review with Narrative Synthesis

**DOI:** 10.3390/jcm13020441

**Published:** 2024-01-13

**Authors:** Wesam A. Debes, Munseef Sadaqa, Zsanett Németh, Ahmad Aldardour, Viktória Prémusz, Márta Hock

**Affiliations:** 1 Doctoral School of Health Sciences, Faculty of Health Sciences, University of Pecs, 7621 Pecs, Hungary; flk0r5@pte.hu (W.A.D.); munseef.sadaqa@etk.pte.hu (M.S.); zsani0008@gmail.com (Z.N.); hock.marta@etk.pte.hu (M.H.); 2 Physical Therapy Department, Rumailah Hospital, Hamad Medical Corporation, Doha P.O. Box 3050, Qatar; aaldardour@hamad.qa; 3 Institute of Physiotherapy and Sports Science, Faculty of Health Sciences, University of Pecs, 7621 Pecs, Hungary; 4 Physical Activity Research Group, Szentágothai Research Centre, 7624 Pecs, Hungary; 5 National Laboratory on Human Reproduction, University of Pecs, 7624 Pecs, Hungary; 6 MTA-PTE Human Reproduction Scientific Research Group, 7624 Pecs, Hungary

**Keywords:** sarcopenic obesity, systematic review, resistance exercise, elderly, women, body composition, functional capacity

## Abstract

Background: Resistance exercise has shown effectiveness in reducing various risk factors related to sarcopenic obesity (SO) compared to other types of exercise, e.g., aerobic exercise. Objective: This systematic review evaluates the effect of resistance exercise on body composition, muscular strength, and functional capacity among older women with sarcopenic obesity aged ≥ 60 years. Methods: This systematic review is registered on PROSPERO (registration No. CRD42023394603) and follows the PRISMA guidelines. The following electronic databases were used to search the literature: Pedro, Cochrane Central Register of Controlled Trials, Embase, PubMed, and Web of Science. We included only RCTs that investigated the effect of resistance exercise on body composition and functional capacity. Two independent reviewers conducted the process of study selection and data extraction. Results: The search strategy retrieved 687 results. One hundred and twenty-six records were deleted as duplicates. Consequently, 534 studies were excluded after the title/abstract assessment. After further detailed evaluation of twenty-seven full texts, seven RCTs were included; all the RCTs examined the isolated effect of resistance exercise in women with sarcopenic obesity. The included studies comprised 306 participants, with an average age of 64 to 72 years. We noticed a trend of improvement in the included studies among the intervention groups compared to the control groups among the different outcomes. The study protocol was inconsistent for the intervention settings, duration, and outcomes. Including a modest number of trials made it impossible to perform data meta-analysis. Conclusions: Heterogeneity among studies regarding training protocols and the outcome measures reported limited robust conclusions. Still, resistance exercise intervention can improve body composition and functional capacity among elderly women with sarcopenic obesity.

## 1. Introduction

Ageing negatively affects every organ in the body; the most noticeable transformations are observed in body composition and mainly involve skeletal muscle, adipose tissue, and bone structure [1]. In 2019, there were about 703 million aged 65 and older worldwide, and this number is expected to reach as much as 1.5 billion by 2050 [2].

In 2010, the European Working Group on Sarcopenia in Older People defined sarcopenia as deterioration in both muscle mass and function (strength or performance) [3]. In 2018, the group met again and updated the previous definition, using low muscle strength as the primary consideration of sarcopenia; muscle strength is considered the most consistent measure related to muscle function. Diagnosis of sarcopenia is more confirmed if it is combined with low muscle quantity or quality. Additionally, if low physical performance is noticed, in addition to low muscle strength and low muscle quantity/quality, sarcopenia is considered severe [4].

Global sarcopenia research has shown significant growth over the past two decades, from 2001 to 2020, with a notable rise in recent years [5]. Estimations of sarcopenia prevalence range from 9.9% to 40.4% among different populations [6]. Moreover, sarcopenia is connected with a decline in functional capacity (i.e., balance performance and mobility) and an increased risk of falls, fractures, and mortality [7,8,9,10]. In addition, sarcopenia has also been associated with increased postoperative complications [11,12]. Therefore, the cumulative evidence supports the belief that sarcopenic individuals represent a vulnerable population to a spectrum of adverse health consequences [13].

Sarcopenic obesity (SO) is defined as reduced lean body mass in excess adiposity [14]. Consequently, it is suggested that SO is predicted to increase as the incidence of obesity rises over time. It is hard to determine the specific prevalence of SO due to the variations of both sarcopenia and obesity. SO is reported to cause many adverse health outcomes, such as reduced physical functioning, including personal care and mobility, and frailty [15,16], in addition to cardiovascular disease (CVD) risk [17]. Furthermore, it has been suggested that individuals with SO have a greater risk of all-cause mortality compared to individuals with either obesity or sarcopenia alone [18].

Previous studies suggested that the male sex is associated with an increased incidence of sarcopenia [19,20] and SO [21]. These results could be understood since the decline in muscle mass with age is insignificant in females compared to men, as muscle mass and function deteriorate significantly through the primary phases of menopause due to the substantial decline in estrogen [22]. Furthermore, distinct sex-specific factors, including hormonal responses and absolute muscle mass, impact the development of age-related muscle disorders [23,24]. Moreover, the steady decline in muscle mass can cause a negative protein balance in the skeletal muscle, with older women exhibiting significantly greater catabolic hormone activity than older men [25].

Exercise is broadly categorised as both preventive and therapeutic [26]. Participating in aerobic exercise can lead to an increase in maximum oxygen uptake, as well as a relative improvement in muscle mass and lower extremity function [27]; however, its impact on enhancing muscle mass in the elderly population is limited [28]. In contrast, resistance exercise shows effectiveness in decreasing multiple risk factors associated with sarcopenic obesity (SO) by increasing muscle strength and growth and enhancing muscle function, in addition to reducing body fat percentage [29,30,31]. This occurs through various mechanisms, such as satellite cell recruitment and the regulation of skeletal muscle growth via activation of the mammalian target of the rapamycin (mTOR) pathway [32]. Additionally, resistance exercise enhances lipolysis and fat oxidation [33]. Consequently, resistance exercise can be considered a primary non-pharmacological intervention for alleviating the consequences of SO [34]. 

In a meta-analysis conducted by Karolina et al., it was concluded that resistance training effectively improved various outcomes among individuals with sarcopenia, including body composition, muscular strength, and functional capacity [35]. Furthermore, a recent systematic review has indicated that resistance training, in particular, has the potential to enhance or preserve physical performance in adults dealing with SO [36].

A previous randomised controlled trial (RCT) demonstrated that participation in resistance exercise enhances functionality and muscular quality among elderly women with sarcopenia. Nevertheless, its impact on muscle growth was limited [37]. Additionally, Chen et al. studied resistance exercise that exhibited enhanced grip strength and knee extensor strength among elderly individuals with sarcopenic obesity compared with alternative training modalities, including aerobic training [26], and an RCT by Kemmler et al. revealed the positive impact of resistance exercise on lean muscle mass and hip/knee extensor strength compared to no intervention with a control group [38]. 

However, there is still a need for a systematic review to summarise the existing evidence about the effect of resistance exercise, specifically among elderly women with SO. Hence, this review provides the opportunity to improve interventions effectively in preventing and treating SO. In the shadow of the information above, this systematic review aims to evaluate the impact of resistance exercise on body composition, muscular strength, and functional capacity in elderly women with SO aged 60 years or more.

## 2. Materials and Methods

The present systematic review adheres to the requirements of the PRISMA 2020 guidelines (Preferred Reporting Items for Systematic Reviews and Meta-Analyses literature search extension) [39]. The review is registered on PROSPERO (registration No. CRD42023394603).

### 2.1. Data Sources and Search Strategies

Two successive searches were independently carried out by two researchers (WD and ZsN). The search was limited to randomised controlled trials in English; the search was conducted on 26 February 2023 for publications published in the following electronic databases: Pedro, Cochrane Central Register of Controlled Trials, Embase, PubMed, and Web of Science. The search was performed using the following terms: Sarcopenia, Muscular Atrophy, Muscle Weakness, Obesity, Weight Lifting, Resistance Training, Strength Training, Female, Women, and Randomized Controlled Trial. The search was limited to articles with the specified terms in their title or abstract. The detailed search strategy is shown in Appendix A. In addition to the initial search, we looked through the reference lists of the articles we had already found. This helped us discover more studies related to the topic. 

We utilised Rayyan (https://www.rayyan.ai/) accessed on 10 March 2023. Rayyan is a web-based tool for systematic review management, to remove duplicates and facilitate the initial screening and selection of articles depending on predefined inclusion and exclusion criteria of our review [40]. 

Following this, two researchers (WD and ZsN) reviewed the titles and abstracts separately. Subsequently, the complete texts of the studies that passed this initial screening were examined to confirm their suitability. Conflicts in opinions between the two researchers were resolved through discussion and agreement or with the input of a third assessor (MH). After the abstract and full-text screening, Cohen’s kappa coefficient (K score) [41] was calculated to weigh the level of agreement between the two reviewers. 

### 2.2. Eligibility Criteria

Following the PICOTS criteria [42], eligible studies were those that were written in English and exclusively focused on women who were ≥60 years of age with sarcopenic obesity (SO). Studies were excluded if they (1) combined two or more interventions other than resistance exercise, (2) had any other study design other than randomised controlled trials (RCTs) (e.g., quasi-experimental studies, cross-sectional studies, and retrospective literature), and (3) patients with severe other complications such as cancer, multiple sclerosis, strokes, cognitive impairment were also excluded from this study. No limitations were placed on publication dates as part of the inclusion criteria. 

### 2.3. Data Extraction

Two independent reviewers extracted data from the selected studies using a predefined data extraction form, including the author, publication year, title, aim and design, number of participants, demographic data, the details of the intervention (such as repetitions and the equipment that were utilised to employ the training, progression of the training, and settings of intervention), outcome measures, results, and limitations.

### 2.4. Risk of Bias Assessment

The risk of bias was assessed independently by two review authors (MS, AD) who were not blind to the trial authors or sources using the recommendations in the Cochrane Handbook for Systematic Reviews of Interventions [43]. We assessed the following domains: bias arising from the randomisation process, bias due to deviations from intended interventions, bias due to missing outcome data, bias in the measurement of the outcome, and bias in the selection of the reported result. Disagreements were resolved through discussion.

## 3. Results

### 3.1. Study Selection and Characteristics

**Figure 1:**
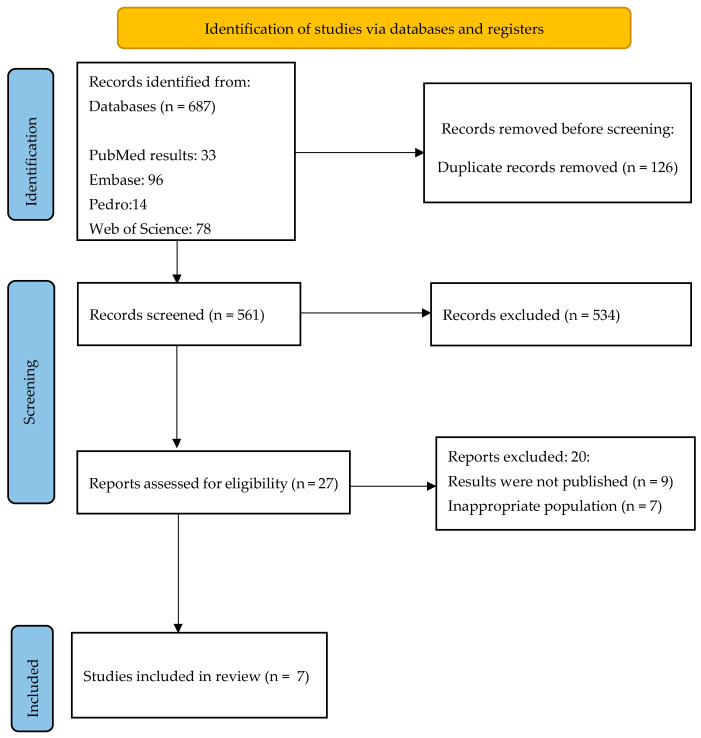
Flowchart of the screened studies.

Among the 687 studies identified, 126 duplicates were removed. Subsequently, through title and abstract screening, 534 additional studies were excluded. The full texts of the remaining twenty-seven studies were evaluated against the inclusion and exclusion criteria, resulting in seven studies meeting the eligibility criteria to be included in the systematic review. Inter-rater reliability between the two reviewers was assessed using a K score, yielding a value of 0.88 at the abstract level and 0.87 at the full-text level, indicating a strong level of agreement between the reviewers. The studies that met the inclusion criteria comprised 306 participants, with 291 individuals remaining until the conclusion of the studies and being subject to analysis for outcome measures. The participants had an average age of 64 to 72 years (Figure 1).

### 3.2. Setting and Training Equipment

The intervention was conducted in rehabilitation departments [44,45], physical therapy departments of universities [46,47], and physical therapy classrooms [48]. One study did not mention where the intervention took place; however, the intervention was most likely conducted in a university laboratory based on the description [49].

Different types of training equipment were used, including resistance machines [47,49], weighted equipment, such as cuffs and vest weights [46], free weights [47,49], elastic bands [44,45,48,50], and body weights [46].

### 3.3. Dosage of Exercise Program 

The duration of exercise interventions for each training session ranged from 30 min to 50 min in one study, depending on which arm [49], and other studies reported that each session was 55 min long [44,48,50], 60 min long [46] and 70 min long [45]. One study did not report the duration of the training sessions [47].

The frequency of the intervention was two sessions per week [46] or three sessions per week [44,45,47,48,49,50]. Regarding the duration of the exercise program, one study reported an exercise program of 10 weeks [46], while the rest had 12-week programs [44,45,47,48,49,50]. The findings from the included studies are summarised in Table 1. 

### 3.4. Results According to the Outcomes

#### 3.4.1. Body Composition Measures 

All of the seven studies included have reported measures related to the participant’s body composition; however, these studies have used various outcomes to assess body composition. Six studies have reported percentage of body fat (BF%), one study has reported improvement in BF% in the three sets group but not in the control or one set grous [49], and another three studies have shown a significant decrease in BF% [47,48,50]; however, two studies did not show significant improvements [44,45].

Four studies reported total skeletal mass. Cunha et al. have reported that skeletal muscle mass in kilograms (kg) in both intervention groups (one set and three sets) has shown improvement compared to pre-training [49]. In addition, another study has significantly improved the exercise group compared to the control group [50]. On the contrary, the other two studies did not reveal significant differences between the exercise and control groups [44,48].

Only two studies have reported body mass index (BMI), and they did not detect any significant difference between the intervention and control groups [45,48]. Moreover, one study has reported waist circumference (WC), hip circumference (HC), and the waist–hip ratio (WHR). This study did not show any significant difference between the groups for WC, HC, or WHR; however, both groups, whey + exercise and placebo + exercise, have shown within-group significant differences for WC and WHR. In addition, no significant within-group difference was observed regarding HC in any of the study arms [47].

Two studies have reported total fat mass (kg). One study showed significant improvement between the groups (Mező [37]), while the other did not reveal any significant difference between the groups [47]. Trunk fat mass was reported in two studies, and neither highlighted any significant difference [47,48]. Trunk muscle mass (kg) was only reported by one study, and it did not reveal any improvement in this outcome [48].

Three articles have studied appendicular lean mass (ALM), and two reported improvement in this outcome [47,50]. Nabuco and colleagues’ study (placebo + exercise) only showed within-group differences [47]. However, one study did not highlight any significant improvement [44].

Two studies reported the lean muscle mass index (LMI) in kg/m^2^. One of them showed improvement [50], whereas the other one did not [44]. In addition, one study reported the appendicular lean mass index (AMI) in kg/m^2^, which resulted in significant improvement in the intervention group compared to the control group. [50]. Only one study reported the skeletal muscle mass index (ALM/height). It showed no significant improvement after three months of intervention [44]. Skeletal muscle mass index percentage (SMI%) was reported by two studies, and the results were not consistent; one study showed a significant difference between groups [50], while the other did not [48]. Lastly, only one study reported total lean soft tissue (LST) and lower LST. This study has highlighted significant improvement compared to pre-intervention for the exercise + placebo group [47]. 

#### 3.4.2. Strength 

Two studies reported grip strength; one study stated a significant increase after the intervention compared to the control [45], while the other did not find any difference [44]. Muscular strength was assessed by the combination of three exercises (load lifted by chest press, load lifted by knee extension, and load lifted by the preacher curl). Two studies reported total strength; one stated that both training groups (one set and three set groups) increased their scores from pre- to post-training, while the control group decreased its score for the same outcome.

Moreover, the group that executed three sets displayed markedly higher muscular strength scores than the group that performed only one set. Still, the one set group scores were significantly higher than the control group [49]. Similarly, the other study reported that both groups, whey + exercise and placebo + exercise, improved their total strength, but there were no differences between the groups [47].

Maximal dynamic strength was evaluated using one repetition maximum (1-RM) in a single RCT. Chest press (kg), knee extension strength (kg), and preacher curl (kg) were used in both groups; whey supplementation + exercise and placebo + exercise showed improvements in post-intervention scores compared to pre-intervention scores, but there were no differences between these two groups [47]. 

Vasconcelos et al. [46], have reported lower limb muscle performance by measuring knee extensor strength in joules, power in watts, and the percentage of fatigue. There were no significant differences between the groups in any of these measurements following the intervention period. The exercise group, however, showed a significant within-group difference in knee muscle power at the 10-week interval.

#### 3.4.3. Functional Capacity 

Five studies reported ten meter walk tests (10 MW) to measure gait speed, and three studies have reported significant differences between the intervention and control groups [44,46,50]; however, two studies have not reported any [45,47]. However, one of these studies reported significant improvement compared to pre-treatment [47].

Three studies have reported timed up and go (TUG) test outcomes to measure lower extremity function, mobility, and fall risk, and two studies have revealed significant improvements in the intervention group compared with the control group after intervention [44,50]; however, one study did not show any difference [45].

Two studies reported functional forward reach (FFR) test outcomes to measure dynamic balance; one study showed significant improvement in FFR scores compared to the control group [50], whereas the other failed to highlight any differences [44].

Two studies reported single leg stance (SLS) tests to measure balance control ability; one of them showed improvement in the intervention group compared to the control group [50], while the other study did not find any difference [44].

Three studies have reported thirty s chair stand test (30 CST) outcomes to measure lower body strength, and all of them reported significant improvement in the intervention group compared to the control group [44,45,50].

Only one study reported raising from a sitting position (RSP), revealing significant improvement between pre- and post-treatment. Still, no significant difference was observed between the whey protein + exercise and placebo + exercise groups [47].

### 3.5. Risk of Bias 

**Figure 2:**
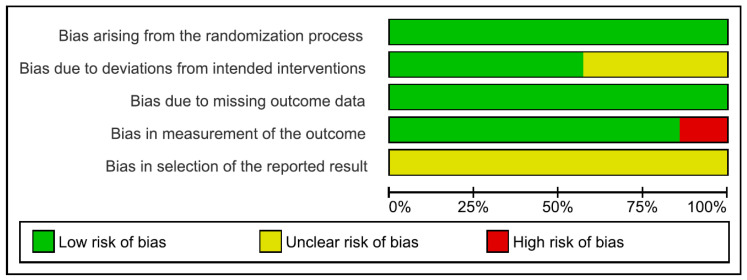
Risk of bias graph.

**Figure 3:**
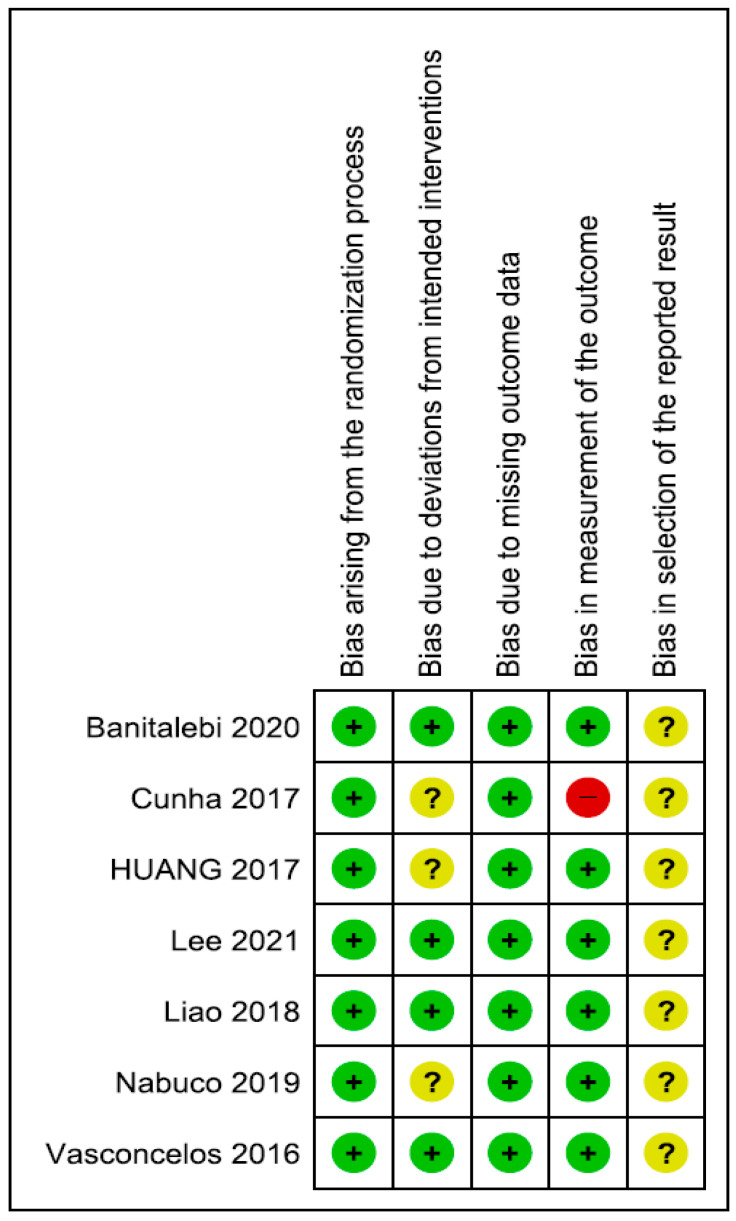
Risk of bias summary. + Low risk of bias, ? Unclear risk of bias, − High risk of bias.

All studies performed a randomisation process with a low risk of bias. Concerning deviations from intended interventions, the risk of bias was low in 57% (4/7) of the included studies and unclear in the remaining 43% (3/7); these studies did not have information on whether appropriate analyses were used to estimate the effect of assignment to intervention. Concerning missing outcome data, the risk of bias was low in all studies. For the measurement of the outcome domain, all studies had a low risk of bias, except one study with a high risk of bias; in this study, there was no information on assessor blinding. Therefore, the assessment could have been influenced by the knowledge of the intervention received. All studies had an unclear risk of bias for the selection of reported results domains since none of the included studies reported that the results produced were analysed following a pre-specified analysis plan. The risk of bias graph and summary are shown in Figure 2 and Figure 3, respectively.

## 4. Discussion

This systematic review aimed to summarise the existing evidence on the effectiveness of resistance exercise on body composition, functional capacity, and muscle strength among women with SO aged ≥60 years. Seven RCTs were included in this systematic review. The study protocol was quite heterogeneous regarding the intervention settings, duration, and outcome measures used to report the changes after conducting resistance exercise training. 

Six RCTs assessed the impact of resistance exercise on anthropometric and body composition measures. BF% emerged as the most reported outcome (six RCTs). In most of the RCTs (67%) (4/6), BF% decreased with resistance exercise intervention. However, two studies did not show any significant changes [44,45]. This inconsistency in the results has also emerged for total skeletal mass, ALM (kg), LMI, and SMI (%). BMI, HC, trunk fat, trunk muscle, and SMI (kg/m^2^) did not show any significant improvement in the exercise group. On the contrary, WC, WHR, total fat mass (kg), AMI (kg/m^2^), total LST, and lower LST have all shown significant improvement in all of the RCTs that reported these outcomes. However previous RCTs reported improvements in thigh lean mass, total body fat, and abdominal fat after resistance exercise intervention compared to the non-training control group, suggesting that resistance exercise improves body composition, and consequently, it is considered an effective treatment for individuals with either sarcopenia or SO [51]. These inconsistencies could be attributed to relatively short periods of intervention of the included RCTs, as all of the included studies were 12 weeks long or less. Still, it is recommended to adhere to resistance exercise for at least 6 months to observe greater improvement in muscle mass for the elderly population [52].

Similar to anthropometric measures and body composition, there was a notable variation in the outcome measures used to assess muscular strength. Five studies reported outcomes related to muscular strength. These measures encompassed a range of parameters, including grip strength (kg) [44,45], chest press (kg) [47], preacher curl (kg) [47], knee extension (kg) [47], and total strength (kg) [47,49], while one study reported knee extensor strength (joules), power (watts), and levels of fatigue (%) [46].

Grip strength showed inconsistent results among the included studies; similarly, the results for muscular strength outcomes were also conflicting among included RCTs. However, total strength, chest press, preacher curl, and knee extension in all included studies showed significant improvement in muscular strength outcomes. In a study by Vasconcelos et al. [46], no significant difference was found between the groups in terms of reported knee extensor strength (joules), power (watts), and levels of fatigue (%). These inconsistencies were observed in the literature. One study reported significant improvement for handgrip and knee extensor strength after resistance exercise training [53], while a meta-analysis by Vlietstra et al. revealed improvement only in knee extensor strength and not in handgrip strength [54]. These differences could be interpreted due to that a short training period does not result in improvement in muscle mass and muscle performance improvement [55]. Moreover, advances in in age and adaptation to resistance exercise that is dependent on mode and dose are worth considering [53,56].

Five studies reported functional capacity, which was assessed by 10 MW, TUG, FFR, SLS, and RSP tests and 30 CST. The 10 MW test was the most reported outcome, and there were five studies that reported it [44,45,46,47,50]. Four of the included studies have shown improvement in the exercise groups for this outcome, except for one RCT [47]. Similarly, improvements in TUG, FFR, and SLS tests were also inconsistent among the included studies. However, the 30 CST and RSP tests have shown improvement in all the included studies. Previous meta-analysis has revealed that resistance exercise has improved gait speed, postural stability, and functional performance during both the early and late stages of sarcopenia [35]. Chen et al.’s meta-analysis showed improvement in TUG and 10 MW, even after a short period of resistance training, and recommended moderate to high intensity (>60% 1-repetition maximum (1RM)) [53]; however, other meta-analyses revealed that high-intensity training (>70–75% 1RM) is more effective in improving functional capacity [56,57]. In our study, the exercise frequency in the included RCTs was two to three sessions per week, which is in line with the recommendation by Chen et al. [53]. Elastic bands are preferred over weight machines for different reasons. First, they might increase the risk of injuries from overexertion, and second, there is a false expectation among the elderly that their use does not require a lot of knowledge [58].

While our review focused on the effects of resistance exercise alone, it is worth considering the possibility that resistance exercise alone may not be sufficient to produce significant improvements in the outcomes of interest, as the impact of protein supplementation combined with resistance exercise was found to be more effective in improving body composition and functionality [59,60]. Moreover, a previous meta-analysis has also suggested that a low-calorie high-protein (LCHP) diet decreases fat mass among elderly individuals with SO [61], and similar findings were found by Yin YH et al. [62]. Other interventions were suggested in the literature. A recent pilot RCT suggested that the administration of oxytocin exhibited potential benefits, manifesting in an increase in whole-body lean mass and a decrease in fat mass compared to the control. Yet, additional investigations with longer follow-up durations and larger sample sizes are necessary to determine the robustness of these results [63]. Moreover, another RCT investigated the impact of high-dose vitamin D supplementation and did not report any significant improvement in sarcopenia or obesity outcomes [64].

Considering the findings in our review, resistance exercise intervention with the recommended dose, duration, and equipment might be effective in improving body composition, anthropometrics, muscular strength, and functional capacity measures among elderly women with SO.

Significant heterogeneity was observed among the included studies, specifically regarding training protocols and outcome measures. Also, it is worth mentioning that the included RCTs have small sample sizes, which can affect the ability to identify significant effects of the intervention in some studies. Due to the reasons above and taking into consideration the limited number of the included RCTs, the authors were unable to conduct a meta-analysis, as performing a meta-analysis using our data is not advisable given the significant risk of bias, as the meta-analysis will prominently mirror the biases present in the individual studies, as suggested by Borenstein et al., who summarised that the effect of studies with different characteristics (mixing apples and oranges) could ignore essential heterogeneity among studies [65], which is the exact case in our review.

These fundamental differences and the scarcity of available evidence highlight the need for further RCTs in this specific group of vulnerable individuals. Such research should explore the factors influencing the effectiveness of resistance exercise and seek to optimise intervention protocols and strategies. The aforementioned constraints prevented a clear conclusion; still, this study highlights important gaps in the research, and this offers the opportunity for future randomised controlled trials (RCTs) to address and refine interventions in the optimal direction. Consequently, these results might enhance precision and efficacy in therapeutic decision-making for treatment protocols of elderly women affected by SO.

Nevertheless, it is important to acknowledge the strengths of this review. Our study employed a rigorous search strategy and followed a strict methodology. Notably, this systematic review represents the first comprehensive investigation of the effect of resistance exercise on functionality among women with SO.

## 5. Conclusions

Heterogeneity among trials and the small number of RCTs affected the conclusions and applicability of conducting a data meta-analysis. However, we noted a pattern of improvement in the majority of the included RCTs concerning body composition, muscular strength, and functional capacity even though effects size and clinical implications cannot be determined precisely. These findings will be supported by future additional high-quality RCTs with more standardised training protocols to confirm the results.

## Figures and Tables

**Table 1:** Complete summary of the key data extracted from the included studies.

Reference	Participants	Duration	Intervention	Outcomes	Summary of Results
Paolo M. Cunha [49]	68 Age (≥60 years)	12 weeks	-Chest press -Horizontal leg press -Seated row -Knee extension -Preacher curl (free weights) -Leg curl -Triceps pushdown -Seated calf raise -Participants of the 1 set per exercise group performed 1 set of 10–15 repetitions maximum for each exercise -Participants of 3 sets per exercise group performed 3 sets of 10–15 repetitions maximum for each exercise	-Body composition was assessed by dual X-ray absorptiometry -Strength was evaluated by 1 repetition maximum testing	-Both training groups increased their scores from pre- to post-training for skeletal muscle mass and total strength. The control group decreased its score for strength. -Only three set groups showed a reduction in relative body fat after the intervention period.
Yu-Hao Lee [44]	27 Age (60–90 years)	12 weeks	Resistance band exercise targeted: shoulders, arms, lower limbs, chest, and abdomen, with 1–2 exercises included for each muscle group	-Body composition was assessed by data obtained from the dual X-ray absorptiometry Functional capacity was assessed by: -FFR -SLS -10 MW -TUG -30 CST Strength was assessed by grip strength	-The exercise group showed improvement for 10 MW, TUG, and 30 CST. -No significant improvement in FFR, SLS, and grip strength was observed. -No significant differences were observed between the study and control groups in terms of changes to body composition.
Karina S. S. Vasconcelos [46]	31 Age (65–80 years)	10 weeks	-Closed and open chain exercise in each leg for posterior, anterior, lateral, and medial muscles of hips and knees -In the first 4 weeks, the resistance exercise program emphasised muscle strengthening and endurance, with concentric and eccentric movements performed at a low speed -From the fifth week, the high-speed “as fast as possible” component was added to the program for concentric movements of exercises -From the seventh to tenth week, concentric and eccentric movements were performed at high speeds -The exercise was only for the legs and hips	-Muscle strength of the lower limbs was measured as the knee extensor strength in joules (J), power in watts (w), and fatigue in percentage (%) using an isokinetic dynamometer -Functional capacity was measured by a 10 MW test	-There were no significant between-group differences for any of the outcomes regarding knee extensors (strength, power, and fatigue). -There was only a significant within-group difference for knee extensor power. -No significant difference regarding the 10 MW test.
Shih-Wei HUANG [48]	35 Age (>60 years)	12 weeks	-Resistance band exercise -One or two types of exercises for training each muscle group, namely the shoulders, arms, lower limbs, chest, and abdomen	Body composition was assessed by data obtained from the dual X-ray absorptiometry	-BF% and total fat mass in the training group showed significant improvement compared to the control group. -No significant difference was found regarding SMI, BMI, trunk fat, trunk muscle mass, and TSM compared to the control group.
Ebrahim Banitalebi [45]	63 Age (65–80 years)	12 weeks	-Resistance band exercise -Exercise included major muscle groups (legs, back, abdomen, chest, shoulder, and arms)	Body composition was assessed by data obtained from the dual X-ray absorptiometry Functional capacities were assessed by: -10 MW -30 CST -TUG Strength was assessed by a grip strength test	-30 CST and grip strength showed significant improvement compared to the control group. -No significant improvement was noticed for 10 MW, TUG, BMI, and BF%.
Liao et al. [50]	56 Age (60–80 years)	12 weeks	-Resistance band exercise -Seated chest press -Seated row -Seated shoulder press -Knee extension -Knee flexion -Hip flexion -Hip extension	Body composition was assessed by data obtained from the dual X-ray absorptiometry. Strength was assessed by: -grip strength Functional capacity was assessed by: -FFR -SLS -10 MW -TUG -30 CST	BF%, TSM, ALM, LMI, AMI, SMI (%), FFR, SLS, 10 MW, TUG, and 30 CST have all shown significant improvement among the experimental group compared to the control group.
Nabuco et al. [47]	26 Age (>60 years)	12 weeks	-Chest press -Horizontal leg press -Seated row -Knee extension -Preacher curl (free weights) -Leg curl -Triceps pushdown -Seated calf raise	Body composition was assessed by data obtained from the dual X-ray absorptiometry Functional capacity assessed by: -10 MW -RSP Muscle strength was assessed by 1 repetition maximum testing	-Total LST, lower LST, ALST, total fat mass, and BF% have all shown more improvement in the whey + exercise group than the placebo + exercise group. -No significant difference between the two groups was found for trunk fat mass, WC, HC, WHR, 10 MW, RSP, knee extension, chest press, preacher curl, and total strength.

10 MW, 10 m walk; 30 CST, 30 s chair stand test; ALM, appendicular lean mass; AMI, appendicular lean mass index; BF%, body fat percentage; BMI, body mass index; FFR, functional forward reach; HC, hip circumference; LMI, lean muscle mass index; LST, Lean soft tissue, ALST, appendicular lean soft tissue, RSP, raising from a sitting position; SLS, single-leg stance; SMI, skeletal muscle mass index; TSM, total skeletal mass (kg); TUG, timed up and go; WC, waist circumference; WHR, waist–hip ratio.

## Data Availability

The original contributions presented in the study are included in the article, and further inquiries can be directed to the corresponding author.

## Abbreviations

1RM	1-repetition maximum
10 MW	10 m walk
30 CST	30 s chair stand test
ALM	Appendicular lean mass
AMI	Appendicular lean mass index
BF%	Body fat percentage
BMI	Body mass index
FFR	Functional forward reach
GS	Grip strength
HC	Hip circumference
LMI	Lean muscle mass index
LST	Lean soft tissue
RCT	Randomised controlled trial
RSP	Raising from a sitting position
SLS	Single-leg stance
SMI	Skeletal muscle mass index
SO	Sarcopenic obesity
TSM	Total skeletal mass (kg)
TUG	Timed up and go
WC	Waist circumference
WHR	Waist–hip ratio

## Appendix A. Search Strategy

**Database**	**Search Strategy**
PubMed	Population: #1 ((((“Sarcopenia”[Mesh]) OR (“Muscular Atrophy”[Mesh])) OR (“Muscle Weakness”[Mesh])) OR (Sarcopenia[Title/Abstract])) OR (sarcopenic[Title/Abstract]) #2 (((((“Obesity”[Mesh]) OR (“Overweight”[Mesh])) OR (obese[Title/Abstract])) OR (obesity[Title/Abstract])) OR (obestic[Title/Abstract])) OR (overweight[Title/Abstract]) #3 Intervention:((((((((((((((((((((((((((((((((((“Weight Lifting”[Mesh])) OR (“Resistance Training”[Mesh])) OR (Resistance Training[Title/Abstract])) OR (resistance exercise[Title/Abstract])) OR (Training, Resistance[Title/Abstract])) OR (Strength Training[Title/Abstract])) OR (Training, Strength[Title/Abstract])) OR (Weight-Lifting Strengthening Program[Title/Abstract])) OR (Strengthening Program, Weight-Lifting[Title/Abstract])) OR (Strengthening Programs, Weight-Lifting[Title/Abstract])) OR (Weight Lifting Strengthening Program[Title/Abstract])) OR (Weight-Lifting Strengthening Programs[Title/Abstract])) OR (Weight-Lifting Exercise Program[Title/Abstract])) OR (Exercise Program, Weight-Lifting[Title/Abstract])) OR (Exercise Programs, Weight-Lifting[Title/Abstract])) OR (Weight Lifting Exercise Program[Title/Abstract])) OR (Weight-Lifting Exercise Programs[Title/Abstract])) OR (Weight-Bearing Strengthening Program[Title/Abstract])) OR (Strengthening Program, Weight-Bearing[Title/Abstract])) OR (Strengthening Programs, Weight-Bearing[Title/Abstract])) OR (Weight Bearing Strengthening Program[Title/Abstract])) OR (Weight-Bearing Strengthening Programs[Title/Abstract])) OR (Weight-Bearing Exercise Program[Title/Abstract])) OR (Exercise Program, Weight-Bearing[Title/Abstract])) OR (Exercise Programs, Weight-Bearing[Title/Abstract])) OR (Weight Bearing Exercise Program[Title/Abstract])) OR (Weight-Bearing Exercise Programs[Title/Abstract])) OR (Lifting, Weight[Title/Abstract])) OR (Liftings, Weight[Title/Abstract])) OR (Weight Liftings[Title/Abstract])) OR (elastic band[Title/Abstract])) OR (body weight training[Title/Abstract])) OR (strengthening exercise[Title/Abstract])) OR (strength exercise[Title/Abstract]) #4 Gender:((((“Female”[Mesh]) OR (“Women”[Mesh])) OR (female[Title/Abstract])) OR (woman[Title/Abstract])) OR (women[Title/Abstract]) #5 Study design: ((((((((((((((((“Single-Blind Method”[Mesh]) OR (“Double-Blind Method”[Mesh])) OR (“Randomized Controlled Trials as Topic”[Mesh])) OR (Randomized Controlled Trial[Publication Type])) OR (“Intention to Treat Analysis”[Mesh])) OR (“Controlled Clinical Trials as Topic”[Mesh])) OR (“Clinical Trials as Topic”[Mesh])) OR (Clinical Trial[Publication Type])) OR (randomized controlled trial[Publication Type])) OR (random*[Title/Abstract])) OR (allocation[Title/Abstract])) OR (random allocation[Title/Abstract])) OR (placebo[Title/Abstract])) OR (single blind[Title/Abstract])) OR (double blind[Title/Abstract])) OR (randomized controlled trial*[Title/Abstract])) OR (RCT[Title/Abstract]) #6 #1 AND #2 AND #3 AND #4 AND #5
Web of Science	#1 (((TS=(Sarcopenia)) OR TS=(sarcopenic)) OR TS=(Muscular Atrophy)) OR TS=(Muscle Weakness) #2 (((TS=(obese)) OR TS=(obesity)) OR TS=(obestic)) OR TS=(overweight) #3 (((((((((((((TS=(Weight Lifting)) OR TS=(Resistance Training)) OR TS=(resistance exercise)) OR TS=(Strength Training)) OR TS=(Weight-Lifting Strengthening Program)) OR TS=(Weight-Lifting Exercise Program)) OR TS=(Weight-Bearing Strengthening Program)) OR TS=(Weight Bearing Exercise Program)) OR TS=(Liftings, Weight)) OR TS=(Lifting, Weight)) OR TS=(elastic band)) OR TS=(body weight training)) OR TS=(strengthening exercise)) OR TS=(strength exercise) #4 ((TS=(female)) OR TS=(woman)) OR TS=(women) #5 ((((((((TS=(Single-Blind Method)) OR TS=(Double-Blind Method)) OR TS=(Randomized Controlled Trial)) OR TS=(Clinical Trials)) OR TS=(random allocation)) OR TS=(placebo)) OR TS=(single blind)) OR TS=(double blind)) OR TS=(RCT) #6 #1 AND #2 AND #3 AND #4 AND #5
Cochrane	#1 MeSH descriptor: [Sarcopenia] explode all trees #2 MeSH descriptor: [Muscular Atrophy] explode all trees #3 MeSH descriptor: [Muscle Weakness] explode all trees #4 (Sarcopenia):ti,ab,kw (Word variations have been searched) #5 (sarcopenic):ti,ab,kw (Word variations have been searched) #6 #1 OR #2 OR #3 OR #4 OR #5 #7 MeSH descriptor: [Obesity] explode all trees #8 MeSH descriptor: [Overweight] explode all trees #9 (obese):ti,ab,kw (Word variations have been searched) #10 (obesity):ti,ab,kw (Word variations have been searched) #11 (obestic):ti,ab,kw (Word variations have been searched) #12 (overweight):ti,ab,kw (Word variations have been searched) #13 #7 OR #8 OR #9 OR #10 OR #11 OR 12 #14 MeSH descriptor: [Weight Lifting] explode all trees #15 MeSH descriptor: [Resistance Training] explode all trees #16 (“resistance training”):ti,ab,kw (Word variations have been searched) #17 (resistance exercise):ti,ab,kw (Word variations have been searched) #18 (Training, Resistance):ti,ab,kw (Word variations have been searched) #19 (Strength Training):ti,ab,kw (Word variations have been searched) #20 (Training, Strength):ti,ab,kw (Word variations have been searched) #21 (Weight-Lifting Strengthening Program):ti,ab,kw (Word variations have been searched) #22 (Strengthening Program, Weight-Lifting):ti,ab,kw (Word variations have been searched) #23 (Strengthening Programs, Weight-Lifting):ti,ab,kw (Word variations have been searched) #24 (Weight Lifting Strengthening Program):ti,ab,kw (Word variations have been searched) #25 (Weight-Lifting Strengthening Programs):ti,ab,kw (Word variations have been searched) #26 (Weight-Lifting Exercise Program):ti,ab,kw (Word variations have been searched) #27 (Exercise Program, Weight-Lifting):ti,ab,kw (Word variations have been searched) #28 (Exercise Programs, Weight-Lifting):ti,ab,kw (Word variations have been searched) #29 (Weight Lifting Exercise Program):ti,ab,kw (Word variations have been searched) #30 (Weight-Lifting Exercise Programs):ti,ab,kw (Word variations have been searched) #31 (Weight-Bearing Strengthening Program):ti,ab,kw (Word variations have been searched) #32 (Strengthening Program, Weight-Bearing):ti,ab,kw (Word variations have been searched) #33 (Strengthening Programs, Weight-Bearing):ti,ab,kw (Word variations have been searched) #34 (Weight Bearing Strengthening Program):ti,ab,kw (Word variations have been searched) #35 (Weight-Bearing Strengthening Programs):ti,ab,kw (Word variations have been searched) #36 (Weight-Bearing Exercise Program):ti,ab,kw (Word variations have been searched) #37 (Exercise Program, Weight-Bearing):ti,ab,kw (Word variations have been searched) #38 (Exercise Programs, Weight-Bearing):ti,ab,kw (Word variations have been searched) #39 (Weight Bearing Exercise Program):ti,ab,kw (Word variations have been searched) #40 (Weight Bearing Exercise Programs):ti,ab,kw (Word variations have been searched) #41 (Lifting, Weight):ti,ab,kw (Word variations have been searched) #42 (Liftings, Weight):ti,ab,kw (Word variations have been searched) #43 (Weight Liftings):ti,ab,kw (Word variations have been searched) #44 (elastic band):ti,ab,kw (Word variations have been searched) #45 (body weight training):ti,ab,kw (Word variations have been searched) #46 (strengthening exercise):ti,ab,kw (Word variations have been searched) #47 (strength exercise):ti,ab,kw (Word variations have been searched) #48 #14 OR #15 OR #16 OR #17 OR #18 OR #19 OR #20 OR #21 OR #22 OR #23 OR #24 OR #25 OR #26 OR #27 OR #28 OR #29 OR #30 OR #31 OR #32 OR #33 OR #34 OR #35 OR #36 OR #37 OR #38 OR #39 OR #40 OR #41 OR #42 OR #43 OR #44 OR #45 OR #46 OR #47 #49 MeSH descriptor: [Female] explode all trees #50 MeSH descriptor: [Women] explode all trees #51 (female):ti,ab,kw (Word variations have been searched) #52 (Woman):ti,ab,kw (Word variations have been searched) #53 (Women):ti,ab,kw (Word variations have been searched) #54 #49 OR #50 OR #51 OR #52 OR #53 #55 #6 AND #13 AND #48 AND #54
Embase	#1. ‘sarcopenia’/exp OR ‘sarcopenia #2. ‘muscle atrophy’/exp OR ‘muscle atrophy’ #3. ‘muscle weakness’/exp OR ‘muscle weakness’ #4. sarcopenia:ab,ti #5. sarcopenic:ab,ti #6. #1 OR #2 OR #3 OR #4 OR #5 #7. ‘obesity’/exp OR ‘obesity’ #8. obese:ab,ti #9. obesity:ab,ti #10. obestic:ab,ti #11. #7 OR #8 OR #9 OR #10 #12. ‘weight lifting’/exp OR ‘weight lifting’ #13. ‘resistance training’/exp OR ‘resistance training’ #14. ‘resistance training’:ab,ti #15. ‘weight lifting’:ab,ti #16. ‘resistance exercise’:ab,ti #17. ‘training, resistance’:ab,ti #18. ‘strength training’:ab,ti #19. ‘training, strength’:ab,ti #20. ‘weight-lifting exercise program’:ab,ti #21. ‘weight-bearing strengthening program’:ab,ti #22. ‘exercise programs, weight-bearing’:ab,ti #23. ‘weight bearing exercise program’:ab,ti #24. ‘weight-bearing exercise programs’:ab,ti #25. ‘lifting, weight’:ab,ti #26. ‘weight liftings’:ab,ti #27. ‘elastic band’:ab,ti #28. ‘body weight training’:ab,ti #29. ‘strengthening exercise’:ab,ti #30. ‘strength exercise’:ab,ti #31. #12 OR #13 OR #14 OR #15 OR #16 OR #17 OR #18 OR#19 OR #20 OR #21 OR #22 OR #23 OR #24 OR #25 OR #26 OR #27 OR #28 OR #29 OR #30 #32. ‘female’/exp OR ‘female’ #33. female:ab,ti #34. woman:ab,ti #35. women:ab,ti #36. #32 OR #33 OR #34 OR #35 #37. ‘single blind procedure’/exp OR ‘single blind procedure’ #38. ‘double blind procedure’/exp OR ‘double blind procedure’ #39. ‘randomized controlled trial (topic)’/exp OR ‘randomized controlled trial (topic)’ #40. ‘randomized controlled trial’/exp OR ‘randomized controlled trial’ #41. ‘intention to treat analysis’/exp OR ‘intention to treat analysis’ #42. ‘controlled clinical trial (topic)’/exp OR ‘controlled clinical trial (topic)’ #43. ‘clinical trial (topic)’/exp OR ‘clinical trial (topic)’ #44. ‘clinical study’:ab,ti #45. ‘randomized controlled trial’:ab,ti #46. random*:ab,ti #47. allocation:ab,ti #48. placebo:ab,ti #49. ‘single blind’:ab,ti #50. ‘double blind’:ab,ti #51. ‘randomized controlled trial*’:ab,ti #52. rct:ab,ti #53. #37 OR #38 OR #39 OR #40 OR #41 OR #42 OR #43 OR #44 OR #45 OR #46 OR #47 OR #48 OR #49 OR #50 OR #51 OR #52 #54. #6 AND #11 AND #31 AND #36 AND #53
Pedro	# Abstract & Title: “Sarcopenic obesity” # Therapy: Strength training # Method: clinical trial # When Searching: Match all search terms (AND)
